# Local Immunosuppressive Microenvironment Enhances Migration of Melanoma Cells to Lungs in DJ-1 Knockout Mice

**DOI:** 10.1371/journal.pone.0115827

**Published:** 2015-02-23

**Authors:** Chia-Hung Chien, Ming-Jen Lee, Houng-Chi Liou, Horng-Huei Liou, Wen-Mei Fu

**Affiliations:** 1 Department of Life Science, College of Life Science, National Taiwan University, Taipei, Taiwan; 2 Department of Neurology, National Taiwan University Hospital, Taipei, Taiwan; 3 Department of Pharmacology, College of Medicine, National Taiwan University, Taipei, Taiwan; 4 Graduate Institute of Brain and Mind Sciences, College of Medicine, National Taiwan University, Taipei, Taiwan; French National Centre for Scientific Research, FRANCE

## Abstract

DJ-1 is an oncoprotein that promotes survival of cancer cells through anti-apoptosis. However, DJ-1 also plays a role in regulating IL-1β expression, and whether inflammatory microenvironment built by dysregulated DJ-1 affects cancer progression is still unclear. This study thus aimed to compare the metastatic abilities of melanoma cells in wild-type (WT) and DJ-1 knockout (KO) mice, and to check whether inflammatory microenvironment built in DJ-1 KO mice plays a role in migration of cancer cells to lungs. First, B16F10 melanoma cells (at 6×10^4^) were injected into the femoral vein of mice, and formation of lung nodules, levels of lung IL-1β and serum cytokines, and accumulation of myeloid-derived suppressor cells (MDSCs) were compared between WT and DJ-1 KO mice. Second, the cancer-bearing mice were treated with an interleukin-1 beta (IL-1β) neutralizing antibody to see whether IL-1β is involved in the cancer migration. Finally, cultured RAW 264.7 macrophage and B16F10 melanoma cells were respectively treated with DJ-1 shRNA and recombinant IL-1β to explore underlying molecular mechanisms. Our results showed that IL-1β enhanced survival and colony formation of cultured melanoma cells, and that IL-1β levels were elevated both in DJ-1 KO mice and in cultured macrophage cells with DJ-1 knockdown. The elevated IL-1β correlated with higher accumulation of immunosuppressive MDSCs and formation of melanoma module in the lung of DJ-1 KO mice, and both can be decreased by treating mice with IL-1β neutralizing antibodies. Taken together, these results indicate that immunosuppressive tissue microenvironment built in DJ-1 KO mice can enhance lung migration of cancer, and IL-1β plays an important role in promoting the cancer migration.

## Introduction

DJ-1, a 20 kD protein belonging to the Thi/PfpI protein superfamily [1], has been regarded as an oncogenic protein to cause certain cancers [2]. Overexpression of DJ-1 has been reported in lung, prostate and breast cancers [3, 4], and DJ-1 appearing in serum can serve as a biomarker for indicating malignancy of breast cancer [5] and melanoma [6]. On the other hand, DJ-1 is linked to early-onset Parkinson’s disease (PD) and loss of DJ-1 can enhance toxin-induced neurotoxicity in DJ-1 knockout (KO) mice [7], and can make cultured neuronal cells more sensitive to oxidative stress. Thus, in terms of oncogenic properties of DJ-1, PD patients with loss of DJ-1 can be predicted to show resistance to cancer. However, PD patients have been reported to have a high risk of getting some cancers, such as melanoma [8, 9], but whether this risk is related to DJ-1 is still unknown. Although DJ-1’s oncogenic effect on cancer cells is clear, its role in tissue microenvironment for cancer development is unknown.

Two oncogenic properties of DJ-1 have been identified. First, DJ-1 is known to serve as a chaperon and anti-oxidative protein to promote survival of cancer cells. It plays an antioxidant role to eliminate hydrogen peroxide through oxidizing 106 cysteine residue to cysteine sulfinic acid against oxidative stress [10]. Second, DJ-1 also possesses anti-apoptotic ability to inhibit cell death through sequestering p53, decreasing expression of Bax, suppressing activation of caspases, or modulating the activity of phosphatase and tensin homolog (PTEN) [3, 11]. However, biochemical impact of DJ-1 molecule has only been evaluated in cancer cells, but not in microenvironment of cancer.

Recently, new evidences have emerged to indicate that DJ-1 is a regulatory protein of inflammation, and its dysregulation can cause proinflammatory response in microglia involved in the development of Parkinson’s disease [12, 13]. In terms of cellular response, knockdown or KO of DJ-1 can sensitize microglia to various inflammatory stimuli to display pro-inflammatory phenotypes [12, 13]. Especially, brain microglia cells with knockdown of DJ-1 has been demonstrated to be highly sensitive to LPS stimulation to release more interleukin-1 beta (IL-1β) [12]. Although the effect of DJ-1 on response of microglia to overexpress IL-1β in brain is evident, its effect on IL-1β levels in cells outside brain is still unclear. Since both macrophage and microglia cells are professional phagocytes with similar characteristics [14, 15], it is interesting to know whether DJ-1 deficiency can also affect IL-1β expression in macrophages. In addition, since IL-1β plays an important role in cancer development, it is also interesting to know whether or not DJ-1 deficiency can impact on cancer development through regulating IL-1β levels.

Local immunosuppressive tissue microenvironment is a critical factor for promoting cancer metastasis and tumor growth [16]. Currently, roles of IL-1β and myeloid-derived suppressor cells (MDSCs) in cancer development have drawn much attention [17, 18]. IL-1β has a biphasic cancer-promoting effect on cancer metastasis, i.e. promoting cancer metastasis either by loss of IL-1β or by excessive IL-1β [18]. In terms of excessive IL-1β, IL-1β can recruit immunosuppressive MDSCs to the lung microenvironment, in which MDSCs can favor cancer development by secreting immunosuppressive cytokines to suppress cytotoxic activities of natural killer, inhibiting T cell proliferation, and enhancing T cell apoptosis. Since DJ-1 deficiency has been demonstrated to up-regulate IL-1β [12], lack or loss of DJ-1 might affect migration of cancer cells to lungs via the IL-1β-regulated MDSCs accumulation in the lung tissue.

Since DJ-1 is a marker for melanoma [6] and PD patients have relatively high risk for melanoma [8, 9], we chose melanoma cells as a model in the present study. To clearly evaluate the microenvironmental effect of DJ-1, we inoculated WT melanoma cells into DJ-1 KO mice, thus excluding the oncogenic effect of DJ-1 in cancer cells and focusing only on the DJ-1 roles in host tissue microenvironment. Our results showed that loss of DJ-1 not only changed IL-1β levels, but also caused an IL-1β-dependent accumulation of MDSCs in the tissue. We also demonstrated that these inflammatory and immunosuppressive changes led to higher migration of cancer cells to lungs in mice.

## Materials and Methods

### Animals, cancer cells, and model of experimental metastasis

WT C57BL/6 mice were provided by the Laboratory Animal Center in National Taiwan University College of Medicine, while DJ-1 KO mice were kindly provided by Dr. Tak Mak’s lab [7]. Male mice were used, at the age between 5 and 6 weeks, and weighed between 20 and 25g. They were housed at room temperature, and allowed to freely access to food and water. All animal experiments were reviewed and approved by the Institutional Animal Care and Use Committee of the National Taiwan University.

B16F10 cells, which belong a murine melanoma cell line of C57BL/6 origin, are poorly immunogenic and do not express pro-IL-1β [19, 20]. Using B16F10 cells as a model can thus exclude immunogenic effect of cancer-derived IL-1β. The cells were obtained from American Type Culture Collection, and were maintained in the humidified incubator (5% CO2, 37°C) in RPMI medium supplemented with 10% heat-inactivated fetal bovine serum (Biological Industries, Kibbutz Beit Haemek, Israel), 100 U/ml penicillin and 0.1 mg/ml streptomycin (Invitrogen, Carlsbad, CA).

To investigate the effects of DJ-1 on tissue microenvironment and migration of cancer cells to lungs, B16F10 cells (at 6×10^4^) were injected into the femoral vein of WT (n = 12) and DJ-1 KO mice (n = 12). Three weeks later, the mice were killed, and nodules in the lung of mice were counted under a dissecting microscope. Lung tissues were then fixed with 4％ paraformaldehyde, embedded in paraffin, cut into sections, and stained with hematoxylin and eosin (H&E) or fluorescent-labeled antibodies. Numbers of lung nodules or fluorescent positive cells were compared between WT and DJ-1 KO mice.

### Stably transfected pool of murine macrophage cells with knockdown of DJ-1

RAW 264.7 murine macrophage cells were maintained in α-MEM medium supplemented with 10% heat-inactivated fetal bovine serum, 100 U/ml penicillin and 0.1 mg/ml streptomycin (Invitrogen, Carlsbad, CA). Knockdown of DJ-1 in the cells were obtained by transfecting the cells with a plasmid vector carrying shRNA which targets DJ-1 transcripts (target Sequence :ATCTGGGTGCACAGAATTTAT), while cells transfected with an empty plasmid vector, i.e. pLKO.1, were used as control. Briefly, RAW 264.7 cells were transfected with the plasmid vectors by using Oligofectamine reagent (Invitrogen, Carlsbad, CA) dissolved in OPTI MEM medium. Six hours after the transfection, the medium was changed back to original α-MEM cultured medium, and puromycin (1 ng/ml) was added into the cultured medium to kill cells without chromosomal integration of gene. A stably transfected pool was established after selection with puromycin, and knockdown of DJ-1 were confirmed by using Western blot analysis.

ELISA and quantitative PCR were performed to compare the expressional levels of IL-1 β in RAW 264.7 cells with (n = 3) and without (n = 3) knockdown of DJ-1. Furthermore, one NF-κB inhibitor and two redox inhibitors were used to treat DJ-1 knockdown RAW 264.7 cells for 6 hours in order to see whether overexpression of IL-1β in the DJ-1 deficiency condition can be counteracted by these inhibitors. The inhibitors used were pyrrolidinedithiocarbamate (PDTC), N-acetyl cysteine (NAC) and Diphenyleneiodonium (DPI) (Sigma, St. Louis, MO, USA).

### Reverse transcription-quantitative polymerase chain reaction

RAW 264.7 murine macrophage cells were lysed by using a guanidinium thiocyanate-phenol-chloroform extraction solution (MDBio Inc., Taipei, Taiwan). Messenger RNA was then converted to complementary DNA (cDNA) by using MMLV RTase (Promega Co., USA), and analyzed by quantitative TagMan real-time polymerase chain reaction (PCR) by using TagMan assay kits (Applied Biosystems, Foster City, CA). The kit contains a pair of predesigned unlabeled PCR primers and one predesigned probe (Mm00434228_m1 for mouse IL-1β; Mm99999915_g1 for GAPDH to serve as a housekeeping-gene control). Finally, real-time PCR was performed by using a StepOne real-time PCR system (ABI, USA), and GAPDH-normalized fold changes of IL-1β were calculated according to the 2^-ΔΔCT^ method [21].

### Western blot analysis

Tissues or cells were lysed in RIPA buffer (150 mM NaCl, 50 mM Tris—HCl, 1 mM EGTA, 1% Nonidet P-40, 0.25% deoxycholate, 1mM sodium fluoride, 50 mM sodium orthovanadate) supplemented with Halt protease inhibitor cocktail (Thermo, IL, USA). Protein concentration was measured by using BCA protein assay kit (Pierce, Rockford, IL) with serially diluted bovine serum albumin as standards. Proteins were separated by SDS-PAGE and transferred to PVDF membranes (Millipore, Billerica, MA). Blotting membranes were then blocked by 5% skim milk dissolved in PBS buffer at room temperature for 1 hour, followed by overnight incubation with primary anti-DJ-1 (1:3000; Enzo Life Sciences, UK) or anti-β-actin (1:10,000; Millipore, MA, USA) antibody at 4°C, and finally by incubation with a HRP-conjugated secondary antibody (1:10,000; Genetex, CA, USA) at room temperature for 1 hour. Protein bands on blots were visualized by using an enhanced chemiluminescence (ECL) detection kit (Thermo, IL, USA), and images of the bands were captured by a chemiluminescence imaging and documentation systems (UVP; Upland, CA, USA).

### Enzyme-linked immunosorbent assay

IL-1β was measured by using the mouse Quantikine enzyme-linked immunosorbent assay (ELISA) kits (R&D Systems, Minneapolis, MN, USA) according to the manufacturer’s instructions. Briefly, samples were pipetted into the microplate wells and incubated for 2 hours at room temperature to let IL-1β be bound by the pre-coated primary monoclonal antibody. After washing away unbound substances, an enzyme-linked polyclonal secondary antibody was added to the wells to form a sandwich complex. A substrate solution was then added to the wells for 30 minutes to yield color. Finally, a stop solution was added, and optical density (O.D.) of each well was measured by using an ELISA reader set at 450 nm. IL-1β concentrations of the samples were then determined by comparing the O.D. of the samples with the standard curve.

### Double-labeling immunohistochemistry

Deeply anesthetized mice were transcardially perfused with 4% saline-diluted paraformaldehyde. The thoracic cavities were opened and lungs were taken out. The lungs were soaked in 4% paraformaldehyde again, trimmed, dehydrated and embedded in paraffin blocks. The paraffin-embedded lungs were then cut into 10 μm thin sections.

For evaluating accumulation of MDSCs, double-labeling immunofluorescent staining of the lung sections was performed. Briefly, the tissue thin sections were deparaffinized and rehydrated, and incubated with 0.05% type XIV protease for antigen retrieval. The thin sections were then incubated with blocking solution (5% BSA and 0.1% Triton X-100 in phosphate-buffered saline) for 1 hour, and finally with a mixture of FITC-labeled anti-mouse Gr-1 antibody (1:500, Biolegend, San Diego, CA) and PE-labeled anti-mouse CD11b antibody (1:500, Biolegend, San Diego, CA). In addition, DAPI counter staining was performed in the same sections to reveal nuclei of cells in the tumor areas. Fluorescent images were captured by the Olympus IX 71 inverted microscope (Olympus, Center Valley, PA, US), and Gr-1^+^/CD11b^+^ cells were cumulatively counted from 9 microscopic fields (100X) randomly selected from WT or DJ-1 KO mice. Average densities of MDSCs, i.e. Gr-1^+^/CD11b^+^ cells, in WT and DJ-1 KO mice were calculated by dividing the total number of cells counted from all fields by the number of fields, and expressed as cell number per field (100X). The densities of MDSCs in WT and DJ-1 KO mice were then statistically compared.

### Multiplex array analysis

Multiplex cytokine arrays were used to compare serum levels of IL-1β and other cytokines in WT (n = 4) and DJ-1 KO mice (n = 4). All serum samples were analyzed by using mouse Cytokine Array Panel A kit (R&D Systems, Minneapolis, MN, USA) according to the manufacturer’s instructions. Briefly, 100 (l serum from a mouse was loaded onto the surface of an array to incubate with the primary antibodies. After washing away unbound proteins, HRP-conjugated secondary antibodies were then applied to the array surface to form sandwich antigen-antibody complexes. After final wash to remove unbound antibodies, protein spots on the array surface were visualized by incubation with ECL substrate. The array images were captured by using a chemiluminescence imaging and documentation systems (UVP, Upland, CA, USA), and abundance of cytokines of both groups in the array images was quantified by using ImageQuant 5.0 software, and normalized against the corresponding cytokines’ levels in the WT group.

### IL-1β neutralization

IL-1β neutralization was done in the animal model by using an IL-1β neutralizing antibody (AF-401-NA; R&D system, Minneapolis, MN, USA) adopted from a previously published literature [18]. The doses of neutralizing antibodies used in different literatures show a dramatic variation, which can be as high as 10 ~ 100 μg for single injection [22–24], or as low as 1 μg for repeated injection [18]. To avoid non-specific immune effect caused by Fc region of antibody, we performed preliminary test and confirmed the lowest effective dose (10 g) for single injection. Briefly, WT (n = 5) and DJ-1 KO (n = 5) mice were treated with a single injection of 10 μg anti-IL-1β neutralizing antibody (AF-401-NA; R&D system, Minneapolis, MN, USA) or 10 μg control immunoglobulins G (AB-108-C; R&D system, Minneapolis, MN, USA) into the femoral vein, following by injection of B16F10 cells (at 6×10^4^) into the same vein after 30 minutes. Three weeks later, the mice were killed, serum IL-1β was measured by using ELISA to confirm neutralization of IL-1β [25], and cancer nodules and MDSCs in lungs of mice were counted.

### Cell viability, proliferation, adhesion and colony formation

MTT conversion and 5-bromo-2-deoxyuridine (BrdU) incorporation assays were performed in 96-wells plates without coating any substances, cell adhesion assay was performed in 96-wells plates pre-coated with 100 (l of 1% BSA (Sigma, St. Louis, MO, USA), 10 μg/ml type I collagen (Millipore, Billerica, MA, USA) or 30 μg/ml fibronectin (Sigma, St. Louis, MO, USA), and colony formation assay was performed in 6-wells plates with 0.7% solid agarose (3 ml) layered on the bottom of each well.

B16F10 cells (5×10^3^ cells/well) were seeded in the 96-well plates and incubated overnight in RPMI medium supplemented with 10% FBS. The medium was then replaced with serum-free medium containing IL-1β (0.2, 2 and 20 ng/ml) for 24 hours. For cell viability MTT assay, the IL-1β containing medium was removed and replaced with MTT solution (0.5 mg/ml, Sigma Aldrich) for 30 minutes. This culture medium was then aspirated, and formazan crystals converted from MTT were dissolved with dimethylsulfoxide (DMSO). The optical densities of the solutions were measured at 570 nm with a microplate reader (BioTek, USA). For cell proliferation assay, a BrdU incorporation-based ELISA (Roche Applied Science, IN, USA) kit was used according to the manufacturer’s instructions. The BrdU incorporation was performed for 4 hours and chemiluminescent signals generated from the ELISA substrate were measured with a microplate luminescent meter (BioTek, USA). To perform cell adhesion assay, B16F10 cells (2×10^4^ cells/well) were seeded on the pre-coated 96-wells plates, incubated for 60 minutes in IL-1β-containing medium (2 and 20 ng/ml IL-1β), and washed two times with PBS to remove free cells. The remaining adhesive cells were then stained with 0.05% crystal violet solution (in 20% methanol), lysed with 10% SDS buffer, and measured by a microplate reader set at 570 nm. To perform colony formation assay, B16F10 cells (2×10^3^ cells/well) were resuspended in 1.5 ml RPMI medium containing 10% FBS and IL-1β (1 or 10 ng/ml), and pre-mixed with 1.5 ml 0.7% cold liquid agarose. The premixed agarose solution was then poured into 6-wells plates which have been layered with 0.7% solid agarose on the well bottom. Once the second cell-containing layer was solidified, 3 ml RPMI medium supplemented with 10% FBS and IL-1β (1 or 10 ng/ml) was then added into the wells as the top layer. The cancer cells were cultured in the soft-agar sandwich for 12 days, and colonies were photographed and counted under inverted microscope.

### Statistics

Statistical analysis between two samples was performed using Student’s t-test. Statistical comparisons of more than two groups were performed using one-way analysis of variance (ANOVA) followed with Bonferroni’s post hoc test. All data were presented as mean ± S.E.M. Difference was considered significant as p < 0.05.

## Results

### Melanoma nodules increase in the lung of DJ-1 KO mice

**Fig 1:**
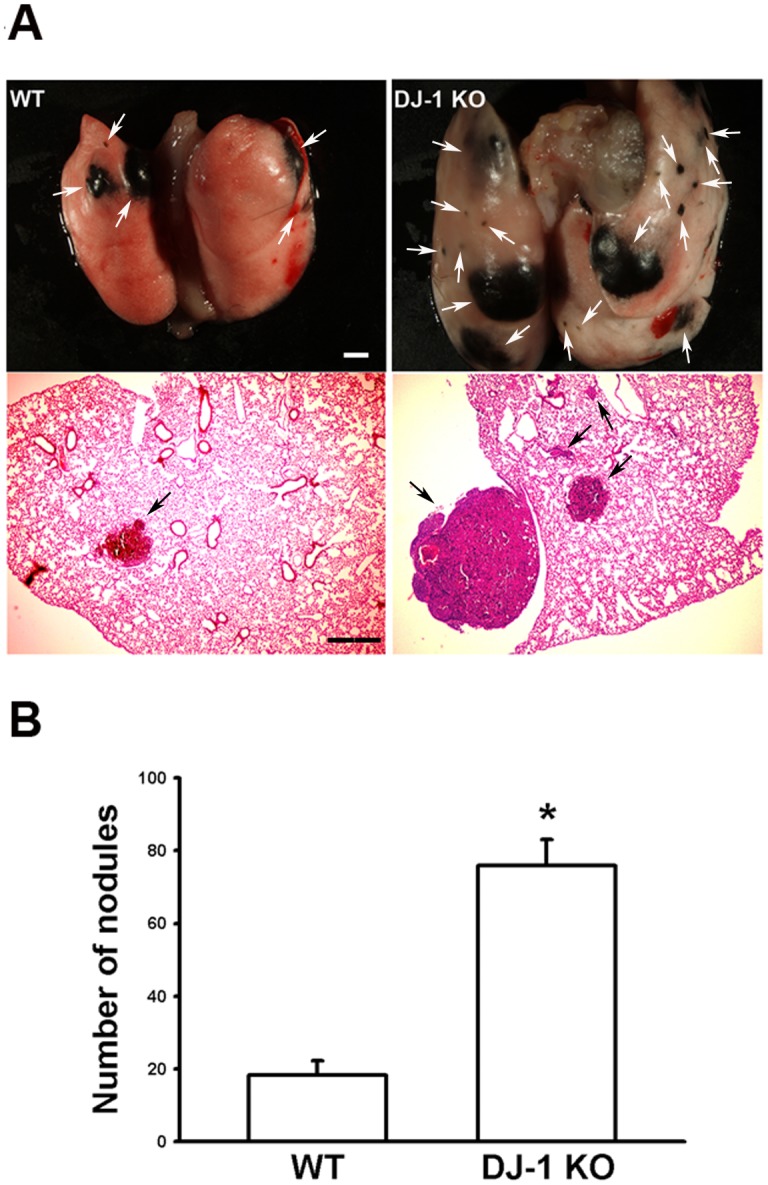
Increase of melanoma lung nodules in DJ-1 KO mice. (A) B16F10 cells (6×10^4^) were intravenously injected into both WT and DJ-1 KO mice. Three weeks later, mice were sacrificed. Gross images showed the melanoma nodules (arrows in upper panel), and histological images showed the tumor masses (arrows in lower panel) in lungs of WT and DJ-1 KO mice. Scale bar: 1 mm for photographs and 0.2 mm for H&E staining. (B) Bar chart showed the summarized results of lung nodule numbers in WT and DJ-1 KO mice. Note that melanoma lung nodules increased in DJ-1 KO mice. Data are presented as mean ± S.E.M. n = 12, for each group, * P < 0.05 compared with WT mice.

To compare the metastatic abilities of murine melanoma cells in DJ-1 KO and WT mice, B16F10 cells (at 6×10^4^) were intravenously injected into mice. As shown in Fig. 1A, more cancer nodules were formed in the lung of DJ-1 KO mice as compared with WT mice 3 weeks later. The mean nodule numbers were 76 ± 7 (n = 12) and 18 ± 4 (n = 12) for DJ-1 KO and WT mice, respectively (Fig. 1B).

### IL-1β levels increase in the lung of DJ-1 KO mice

**Fig 2:**
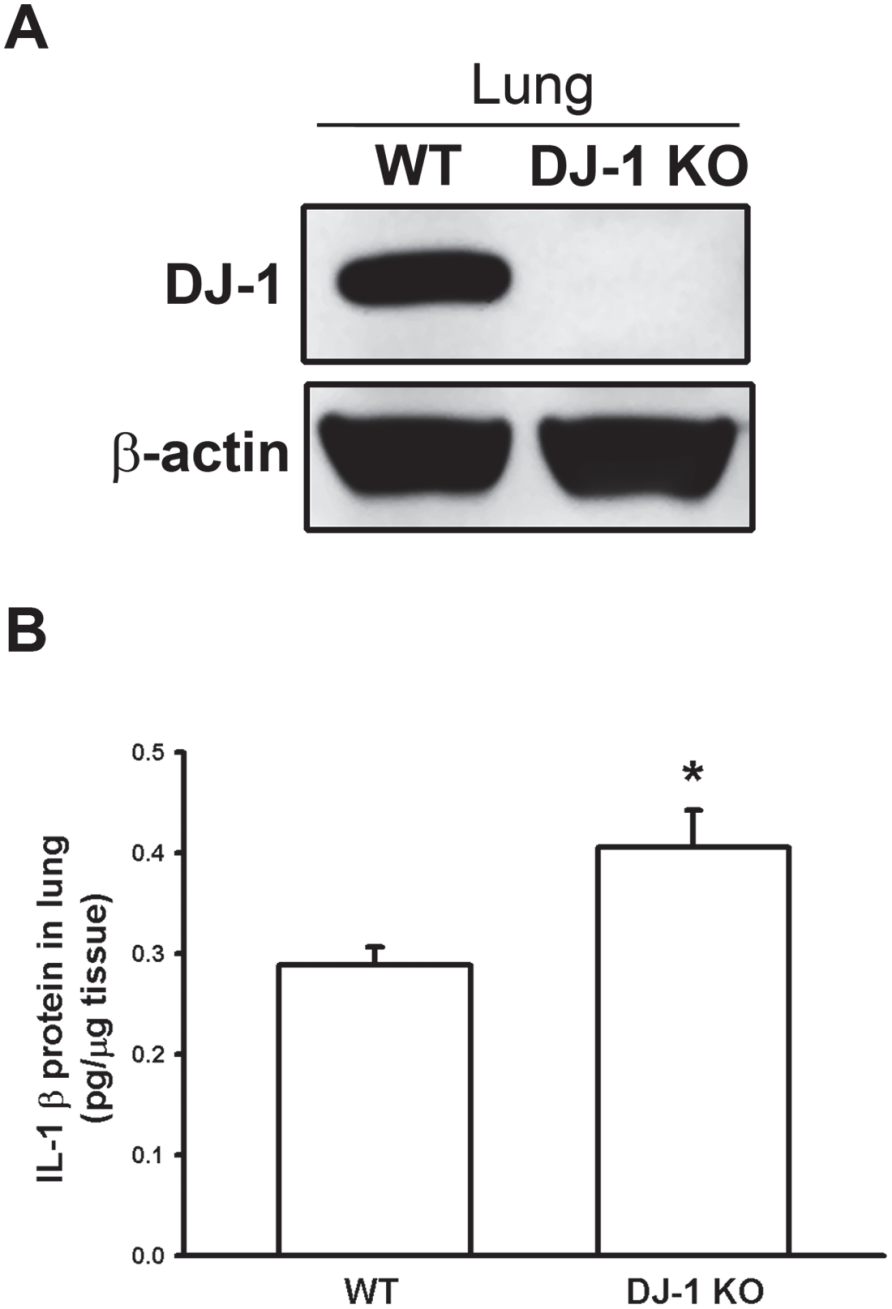
Increase of IL-1β expression in lung tissues in DJ-1 KO mice. (A) The lung tissues were isolated for Western blotting. Western blots showed that DJ-1 expression was totally abolished in DJ-1 KO mice. (B) ELISA measurement showed that IL-1β expression increased in lungs of DJ-1 KO mice in comparison with WT mice. Data are presented as mean ± S.E.M. n = 5 for each group, * p < 0.05 compared with WT mice.

DJ-1 and IL-1β levels were measured by using Western blotting and ELISA, respectively. As shown in Fig. 2A, complete loss of DJ-1 was verified in the lung tissue in DJ-1 KO mice as compared with WT mice (n = 5). In contrast, levels of IL-1β were significantly up-regulated in the lung tissue in DJ-1 KO mice (n = 5) (Fig. 2B). Our data are consistent with previous finding in microglia cells that DJ-1 deficiency can up-regulate IL-1β [12].

### Change of serum levels of IL-1β and other cytokines in DJ-1 KO mice

**Fig 3:**
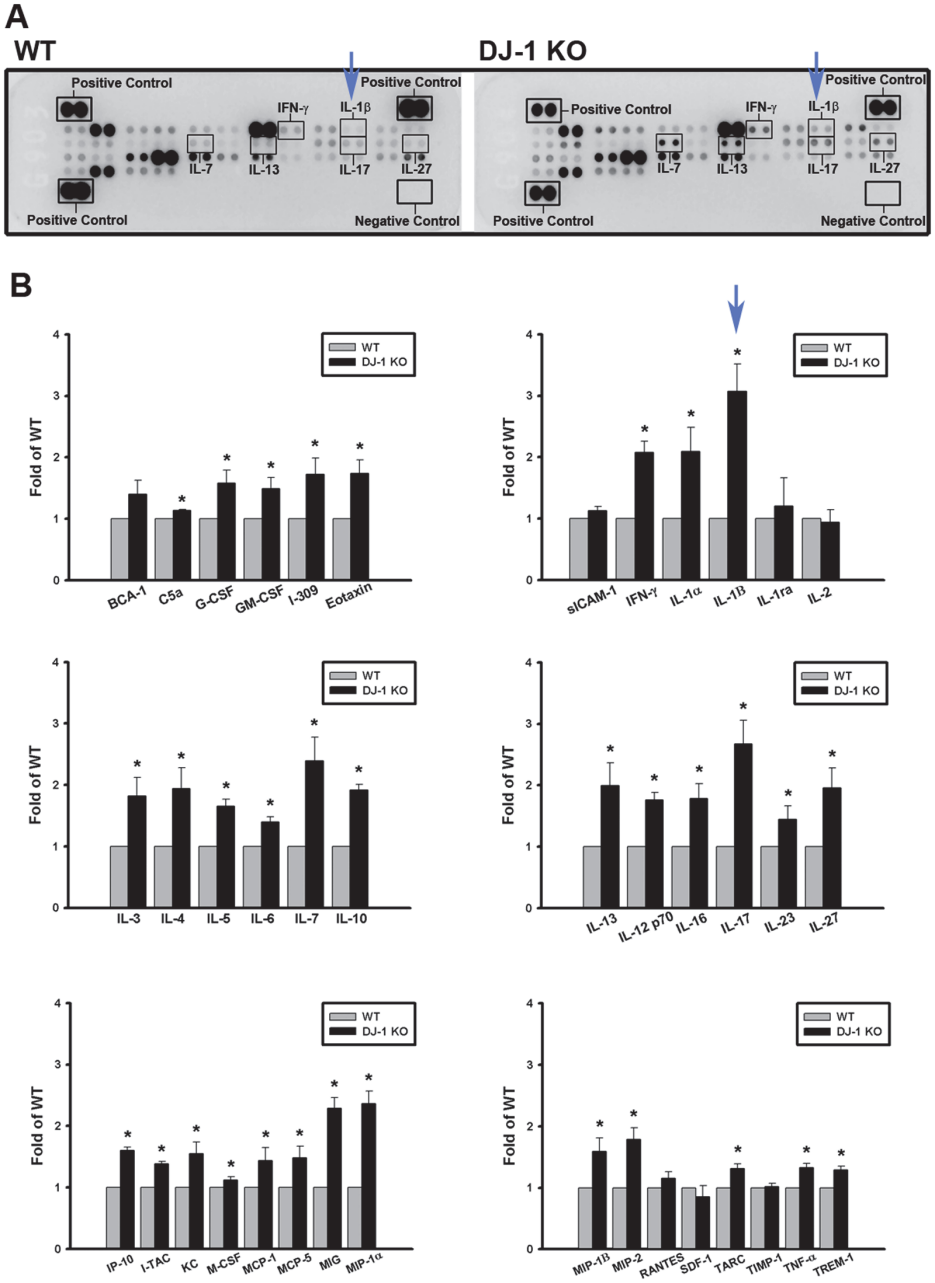
Increase of serum levels of IL-1β and other cytokines in DJ-1 KO mice. (A) Representative protein array images displayed the serum cytokine profiles in WT (left) and DJ-1 KO (right) mice. Each protein was measured in duplicate on an array. Positions of IL-1β spots are indicated by blue arrows. (B) Bar charts showed the fold differences of proteins in cytokine arrays between WT and DJ-1 KO mice. The data were presented as mean ± S.E.M., and IL-1β bars are indicated by the blue arrow. All data were normalized to respective protein spots in WT groups. n = 4 for each group, * p < 0.05 compared with WT mice.

We then performed cytokine-array analysis to compare the serum levels of IL-1β and other inflammatory cytokines between WT and DJ-1 KO mice. The array images showed that serum IL-1β and several cytokines were significantly elevated in DJ-1 KO mice (n = 4) as compared with WT mice (n = 4) (Fig. 3A).

The statistical analysis further revealed that the serum levels of IL-1β level in DJ-1 KO mice have the most significant change (3.07 fold) as compared with WT mice (blue arrows in Fig. 3A and 3B). The mean serum levels of several other cytokines or chemokines in DJ-1 KO mice had more than 2-fold increase (Fig. 3B), including IFN-γ (2.08 fold), IL1-α (2.09 fold), IL-7 (2.39 fold), IL-17 (2.67 fold), MIG (2.29 fold) and MIP-1α (2.37 fold). These results suggest that loss of DJ-1 can increase systemic inflammatory tone in the animals.

### IL-1β neutralizing antibody antagonizes the increase of melanoma lung nodules in DJ-1 KO mice

**Fig 4:**
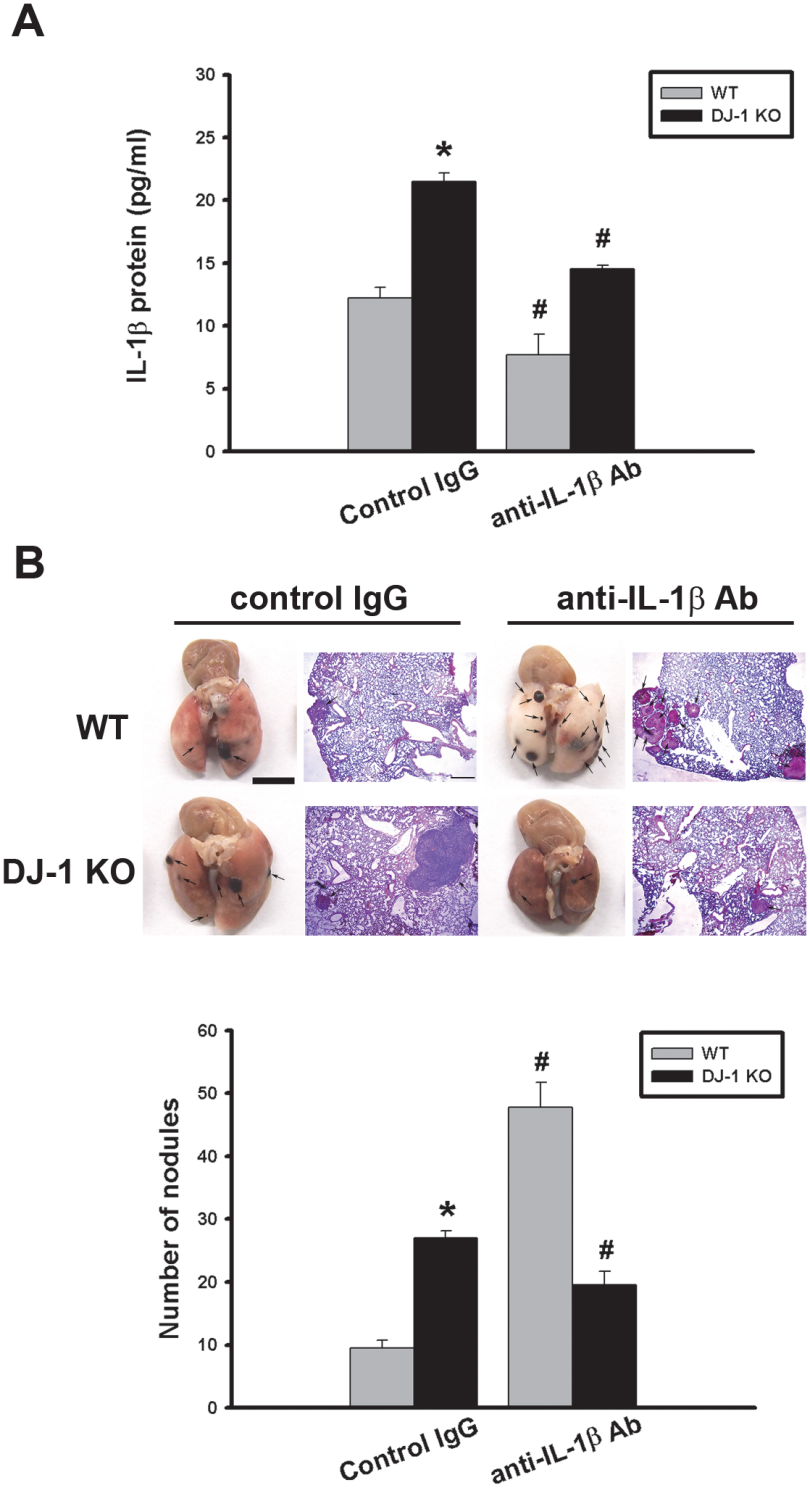
Differential effects of IL-1β neutralization on melanoma lung nodules in WT and DJ-1 KO mice. (A) IL-1β antibody (10μg) or control IgG was intravenously injected into mice once. The melanoma cells (6×10^4^) were intravenously injected 30 minutes later. The mice were sacrificed three weeks later. Bar chart showed that the anti-IL-1β neutralizing antibody can effectively lower serum IL-1β levels as compared with control IgG in both WT and DJ-1 KO mice. Note that serum IL-1β levels were higher in DJ-1 KO mice than those in WT mice. (B) Inhibition of IL-1β neutralization on lung nodules formation in DJ-1 KO mice. Upper panels: melanoma nodules in lungs of WT (top) and KO (bottom) mice treated with control IgG (left) or neutralizing anti-IL-1β antibody (right). For each experimental condition, the gross and microscopic morphologies of tumor nodules are indicated by arrows, and are shown at left and right sites, respectively. Scale bars: 0.5 mm for lung photographs and 0.5 mm for H&E staining. Lower panel: Bar chart showed the summarized results of lung nodule numbers in WT and DJ-1 KO mice. Note that IL-1β neutralization enhanced and suppressed melanoma nodules formation in WT and DJ-1 KO mice, respectively. Data are presented as mean ± S.E.M. n = 5 for each group, * p < 0.05 compared with control IgG-treated WT mice, # p<0.05 compared with respective control IgG in WT and DJ-1 KO mice.

Since IL-1β has a biphasic effect on cancer metastasis when it is either insufficient or excessive [18, 26, 27], we then checked effects of IL-1β neutralization on migration of melanoma cells to lungs in both WT and DJ-1 KO mice. As shown in Fig. 4A, single intravenous injection of anti-IL-1β neutralizing antibody (10 μg) significantly reduced the serum level of IL-1β in comparison with the equivalent dose of control immunoglobulins G (IgG) in both WT (n = 5) and KO (n = 5) mice (F = 29.883, P < 0.05), indicating successful neutralization in both groups.

Consistent with previously published finding [18], injection of IL-1β-neutralizing antibodies greatly increased the formation of lung melanoma nodules in WT mice (gray bars in Fig. 4B), which confirmed that insufficient IL-1β favored cancer development [18]. In the contrary, blockade of IL-1β by injecting with neutralizing antibodies antagonized the nodule formation in DJ-1 KO mice (black bars in Fig. 4B), indicating that IL-1β signaling is involved in the DJ-1 deficiency-enhanced migration of cancer cells to lungs (n = 5 for each group, F = 44.622, P < 0.05).

### IL-1β neutralizing antibody antagonizes the increased accumulation of MDSCs in the lung of DJ-1 KO mice

MDSCs are immunosuppressive cells defined as CD11b^+^ / Gr-1^+^ cells and accumulation of MDSCs can be estimated by double immunofluorescent staining of two markers. To examine whether IL-1β builds an immunosuppressive microenvironment, we then checked the effect of IL-1β neutralization on the accumulation of MDSCs in lungs.

**Fig 5:**
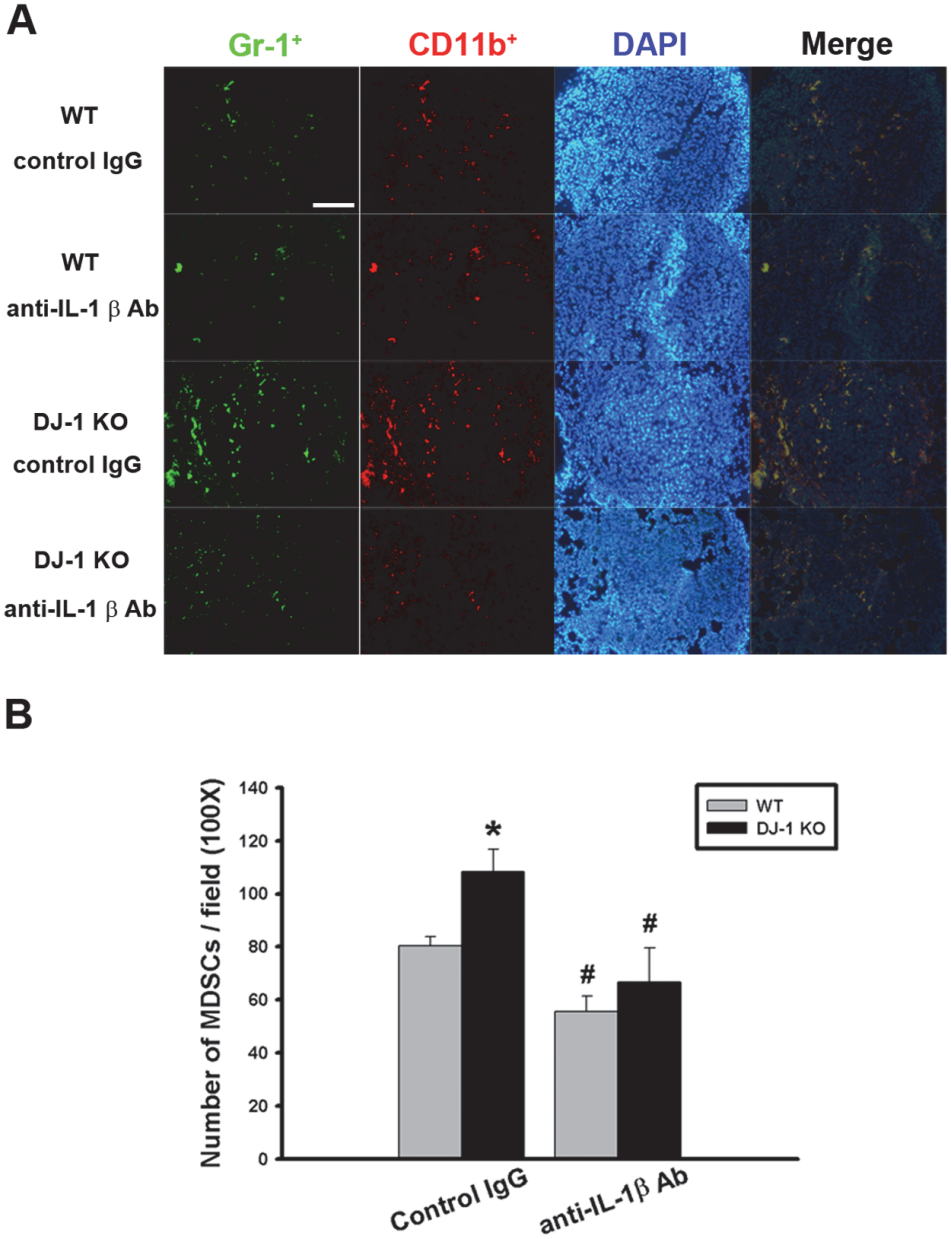
Effects of IL-1β neutralization on the accumulation of MDSCs in lungs. (A) Immunofluorescent photos showed that Gr-1+ (green)/CD11b+ (red) cells indicate MDSCs and increased in lungs of DJ-1 KO mice. Treatment with anti-IL-1β neutralizing antibody antagonized the accumulation of MDSCs. DAPI was used to stain nuclei. (B) Bar chart showed the summarized results of MDSCs in lungs of WT and DJ-1 KO mice. Note that the neutralizing antibody significantly reduced the number of MDSCs accumulated in lungs. n = 9 for each group, * p < 0.05 compared with control IgG-treated WT mice, # p<0.05 compared with respective control IgG in WT and DJ-1 KO mice.

Our results showed that number of CD11b^+^ / Gr-1^+^ cells were significantly higher in the lung of control IgG-treated DJ-1 KO mice as compared with control IgG-treated WT mice (Fig. 5A), indicating an immunosuppressive microenvironment was established in the lung of DJ-1 KO mice, which is known to favor the growth of cancer [28]. Our data further demonstrated that accumulation of MDSCs in lungs was significantly antagonized by the treatment of IL-1β neutralizing antibodies (Fig. 5A), which is consistent with previous reports that IL-1β plays a key role in MDSCs accumulation [17, 18]. Moreover, decrease of MDSCs accumulation by IL-1β neutralizing antibodies was more pronounced in DJ-1 KO mice (n = 9) than WT mice (n = 9) (Fig. 5B) (F = 8.576, P < 0.05), suggesting that massive accumulation of MDSCs in KO mice might be caused by excessive IL-1β in the DJ-1 deficient condition.

### Knockdown of DJ-1 increases IL-1β expression in macrophages

**Fig 6:**
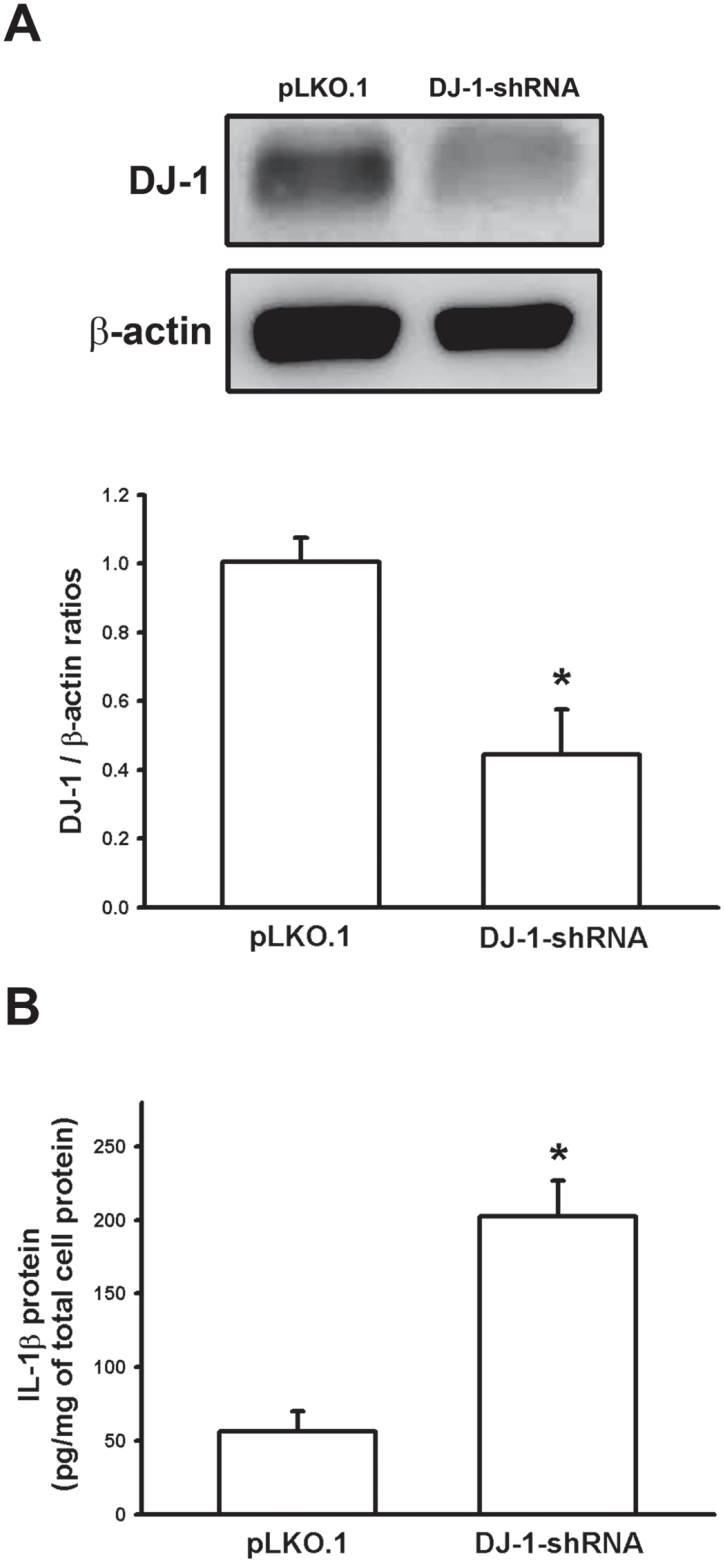
Increase of IL-1β expression in DJ-1 knockdown macrophages. (A) Western blots showed that DJ-1 was knockdowned in RAW 264.7 cells. Upper panel: representative blots of DJ-1 and β-actin in cells stably transfected with empty plasmids (pLKO.1) or plasmids encoding DJ-1-shRNA. Lower panel: bar chart showed the summarized results of Western blot. (B) ELISA analysis showed the up-regulation of IL-1β protein expression in RAW 264.7 cells stably transfected with DJ-1-shRNA plasmids as compared to cells transfected with pLKO.1 plasmids. Data are presented as mean ± S.E.M. n = 3 for each group, * p < 0.05 compared with pLKO.1 groups.

Although DJ-1 deficiency has been demonstrated to increase IL-1β expression in glia cells, it is still unknown whether or not DJ-1 deficiency can also enhance IL-1β expression in other types of cells. Macrophages are a main source of IL-1β in serum or tissues, we thus used macrophages as a cell model to examine effects of DJ-1 knockdown on IL-1β expression. As demonstrated by Western blotting, DJ-1 protein expression was significantly decreased in the cell pool stably transfected with shRNA-plasmids (DJ-1-shRNA) as compared with the pool of empty plasmids (pLKO.1), indicating that stable knockdown of DJ-1 has been successfully established in RAW 264.7 cells (Fig. 6A).

We then compared the expression of IL-1β between the pLKO.1 and DJ-1-shRNA groups, and it was found that levels of IL-1β were significantly enhanced in DJ-1-shRNA group (n = 3) as compared with pLKO.1 group (n = 3) (Fig. 6B), indicating that DJ-1 deficiency can enhance IL-1β expression in macrophages.

### DJ-1 increases IL-1β expression through NF-κB but not redox signaling

**Fig 7:**
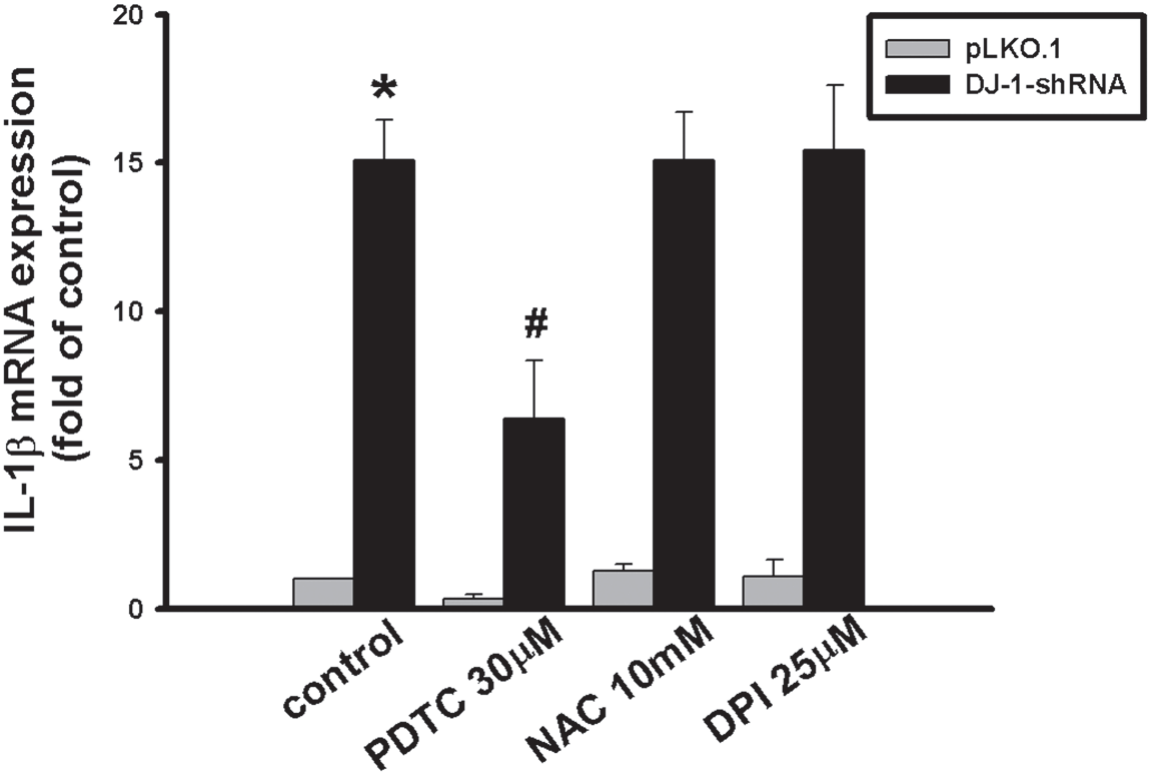
Blockage of DJ-1 knockdown-induced IL-1β overexpression by NF-κB inhibitor in RAW cells. Quantitative RT-PCR showed that there was a higher level of IL-1β transcript in RAW 264.7 cells with DJ-1 knockdown as compared to control cells, which was partially reversed by treatment with NF-κB inhibitors PDTC, but not by redox inhibitors NAC and DPI. Data were presented as mean ± S.E.M. n = 3 for each group, * p < 0.05 compared with pLKO.1 control group, # p< 0.05 compared with DJ-1-shRNA control group.

Since DJ-1 is a redox sensor which can transduce redox signals [29] and IL-1 in myeloid and lymphoid cells can be regulated by NF-**κ**B [30, 31], DJ-1 may thus increase IL-1β expression through redox and/or NF-κB signaling. Blockade of these two pathways was performed to see if the effect of DJ-1 deficiency can be antagonized. As shown in Fig. 7, the enhanced expression of IL-1β in DJ-1 knockdown condition can only be suppressed by PDTC (F = 40.812, P < 0.05), which is a NF-κB inhibitor, but not by redox inhibitors of NAC and DPI. These results indicate that DJ-1 regulates IL-1β expression through NF-κB signaling, but not via redox signaling.

### IL-1β increases cell viability, proliferation, adhesion, and colony formation in melanoma cells

**Fig 8:**
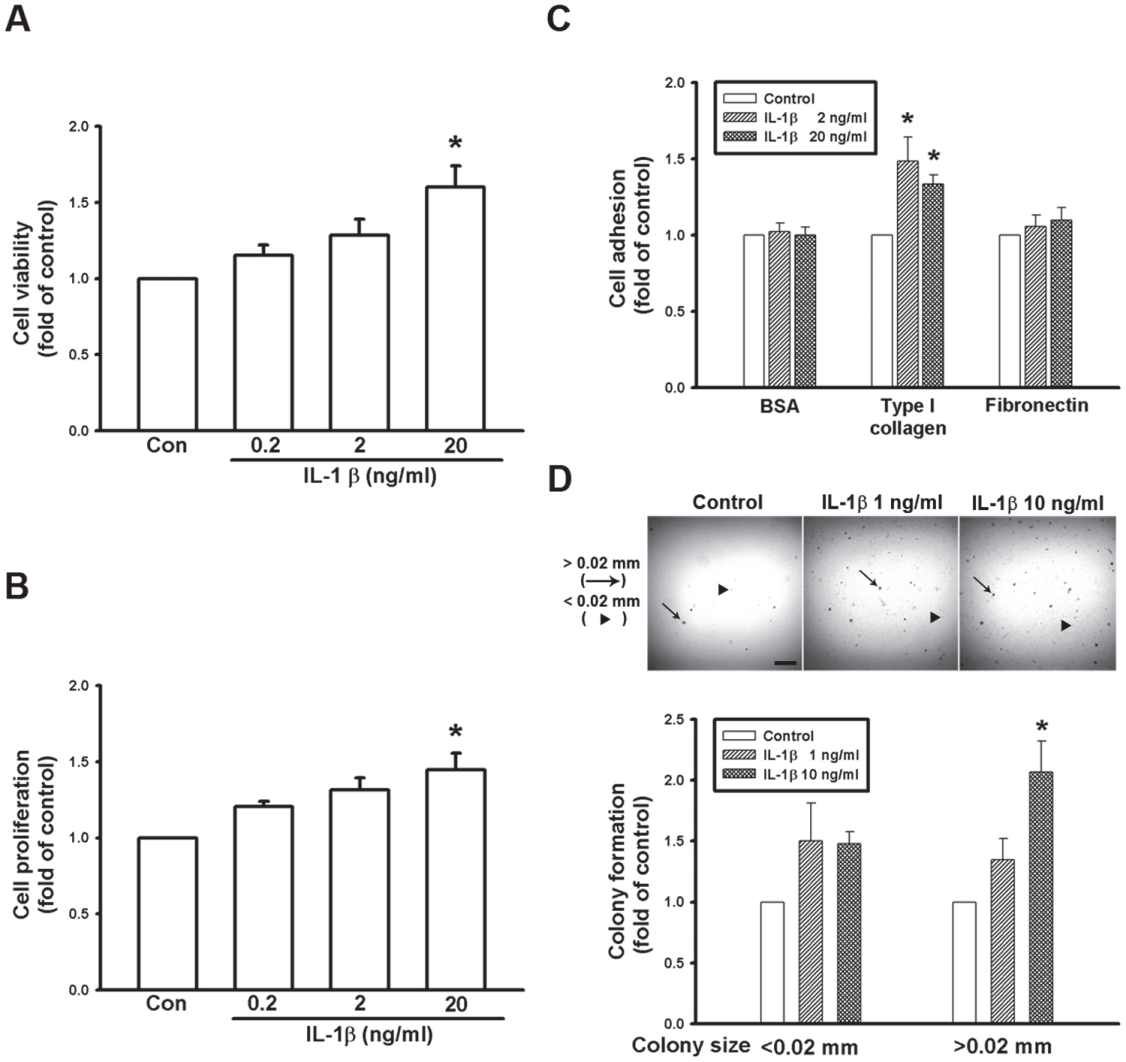
Effects of IL-1β on cell viability, proliferation, adhesion and colony formation. (A) Cell viability was evaluated using MTT assay. Note that treatment of IL-1β (20 ng/ml) increased cell viability of melanoma cells. (B) Cell proliferation was measured using Brdu incorporation method. Note that treatment of IL-1β (20 ng/ml) enhanced cell proliferation. (C) Effects of IL-1β on cell adhesive ability. Note that IL-1β (2 and 20 ng/ml) can increase adhesion of melanoma cells on type I collagen, but not on albumin and fibronectin. (D) Colony formation of melanoma cells in soft agar with or without addition of IL-1β. Upper panel: microscopic photos showed the colony of large sizes (> 0.02mm, arrow) and small sizes (< 0.02mm, arrow head). Lower panel: bar chart showed that IL-1β increased the number of colony with large sizes. Data are presented as mean ± S.E.M. n = 3 for each group, * p < 0.05 compared with control groups.

IL-1β has been known to have a wide range of biological activities, not only on recruiting MDSCs (Fig. 5), but also directly on various characteristics of cancer cells [27]. To explore effects of IL-1β on various metastatic characteristics of melanoma cells, we examined cell viability, proliferation, adhesion ability, and colony formation of IL-1β-treated B16F10 cells. Our data showed that both conversion of MTT to formazan (Fig. 8A) and incorporation of BrdU (Fig. 8B) were significantly increased in IL-1β-treated B16F10 cells (F = 7.851 and 5.337 respectively, P < 0.05) at concentration of 20 ng/ml, indicating that IL-1β can directly increase cell viability and proliferation. In addition, cell adhesion to type I collagen (Fig. 8C), which is the main component of lung extracellular matrix [32], and colony formation of cancer cell in agarose gels (Fig. 8D) were also significantly increased in IL-1β-treated B16F10 cells (F = 11.251 and 9.328 respectively, P < 0.05) as compared with the control cells, suggesting that IL-1β can directly enhance metastatic ability of melanoma in the severe inflammatory condition.

## Discussion

So far, roles of DJ-1 in regulating IL-1β expression and building inflammatory microenvironment for cancer development are still unclear. In the present study, we demonstrated that migration of melanoma cells to lungs was greatly increased in DJ-1 KO mice as compared with WT mice (Fig. 1). The increased migration was correlated to the up-regulated levels of IL-1β in lung tissues and serum (Figs. 2B and 3), as well as correlated to the increased accumulation of MDSCs in lungs (Fig. 5). We also demonstrated that blockade of IL-1β with neutralizing antibodies can reduce the formation of melanoma nodules (Fig. 4B, black bars) and lower MDSCs accumulation (Fig. 5) in lung tissues in DJ-1 KO mice, suggesting that DJ-1 loss-induced MDSCs accumulation and tumor development in lungs are IL-1β dependent. Using cultured cells as models, we further demonstrated that DJ-1 deficiency can cause the overproduction of IL-1β in macrophages through NF-κB signaling (Figs. 6 and 7). In addition, IL-1β can directly act on melanoma cells to promote their survival, proliferation, adhesion and colony formation (Fig. 8).

Migration of cancer cells to lungs after tail injection of them depend on the interaction of cancer cells with tissue or serum microenvironment, in which inflammatory cells play a key role for cancer development [28, 33, 34]. In terms of tissue microenvironment, our results revealed that IL-1β was involved in recruiting MDSCs to lung tissues and building an immune suppressive microenvironment in DJ-1 KO mice. The accumulation of MDSCs in lungs can be antagonized by IL-1β neutralizing antibody. This finding is consistent with previous reports [17, 18], which show that overexpression of IL-1β can induce spontaneous gastric cancer by mobilizing MDSCs, and microenvironment-derived IL-1β can promote the lung metastasis of epithelial tumor cells through MDSCs accumulation. Although IL-1β is known to be expressed in certain cancer cells, we have excluded the role of tumor-derived IL-1β in building the immunosuppressive microenvironment, as IL-1β is not detectable in B16F10 melanoma cells in WT and KO mice (data not shown). In contrast, our results revealed that shRNA-mediated suppression of DJ-1 can increase IL-1β expression in macrophages, suggesting that the excessive IL-1β in DJ-1 KO mice might come from macrophages to serve as a paracrine to recruit MDSCs. Since MDSCs are a heterogeneous population of early myeloid progenitors which can also differentiate into macrophages, it cannot be ruled out that MDSCs with DJ-1 KO can also express IL-1β to serve as autocrine to recruit more MDSCs, and this possibility needs further investigation. Although IL-1β has been known to activate NF-κB signaling in MDSCs to affect tumor progression [17, 18], IL-1β has recently been reported to influence B16 tumor progression via STAT1 signaling [35]. The IL-1β induced signal transduction in MDSCs also needs further investigation.

In addition to tissue IL-1β, our results also revealed that serum IL-1β was also dramatically altered in DJ-1 KO mice, together with changes of other serum cytokines. The minimal effective IL-1β concentration (0.2–2 ng/ml) to enhance proliferation, adhesion and colony formation of cultured melanoma cells was higher than the IL-1β concentration in serum (i.e. 22 pg/ml in KO and 12 pg/ml in WT mice). This observation is consistent with previous reports that effective concentration of interleukin to increase malignant potentials of cancer cells is higher than its serum level in mice [36, 37], and suggests that the direct effect of IL-1β on melanoma cells should be more obvious in the severe inflammatory condition. The effect of circulating IL-1β in serum on migration of cancer cells to lungs has been supported by another report, which shows that injection of recombinant IL-1β into blood can promote experimental lung metastasis of tumor [18]. So far, DJ-1-related literatures all focus on the behaviors of cancer cells with knockdown or KO of DJ-1. To the best of our knowledge, the present study is the first to examine the metastatic behavior of WT cancer cells in the DJ-1 KO microenvironment of tissue. This strategy can exclude the oncogenic effect of DJ-1 in cancer cells, and can focus only on the DJ-1 roles in the microenvironment of cancer.

**Fig 9:**
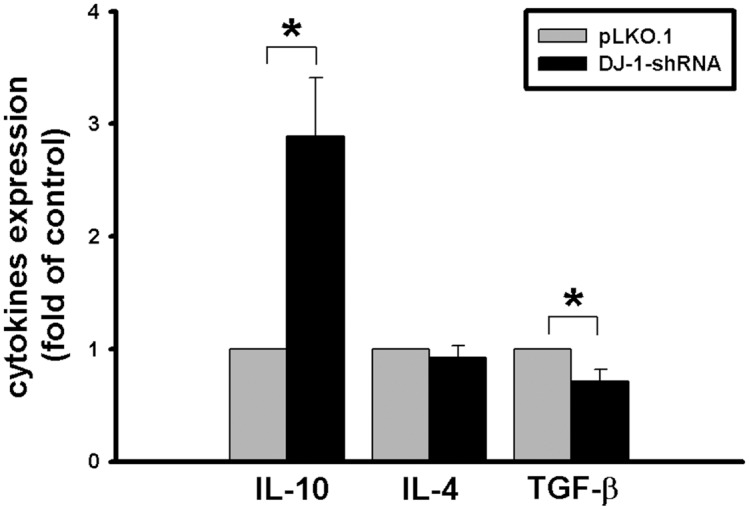
Expressional profiles of immunosuppressive cytokines IL-4, IL-10, and TGF-β in macrophages with and without DJ-1 knockdown. Bar charts showed the fold changes of cytokine expression in macrophages without (pLKO.1) and with (DJ-1 shRNA) knockdown of DJ-1. The data were presented as mean ± S.E.M. (n = 3 for each group). All data were normalized to respective cytokine levels in pLKO.1 groups to calculate fold change (* p < 0.05).

**Fig 10:**
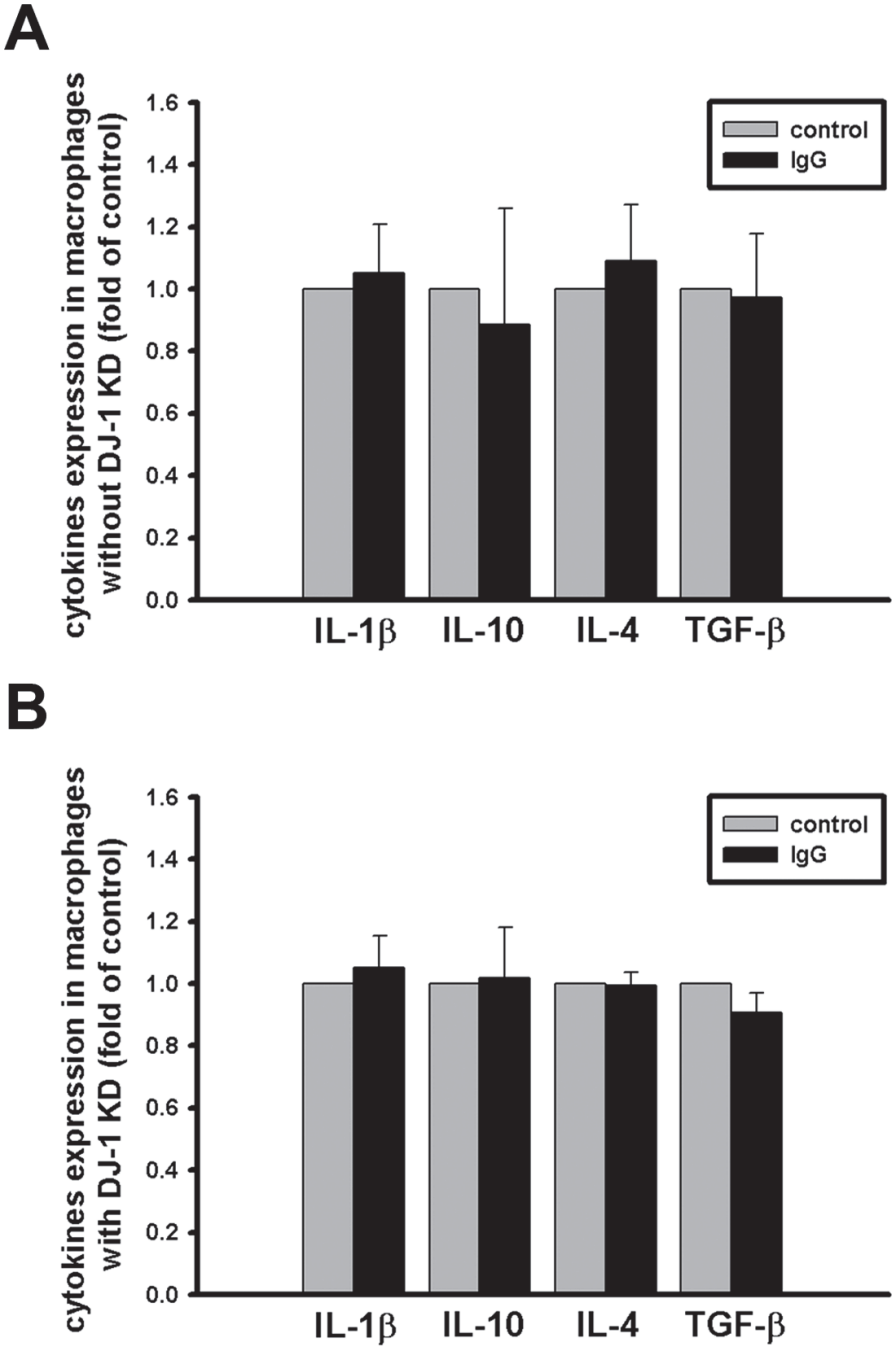
Effects of the isotype-control antibody on expression of immunosuppressive cytokines in macrophages. Bar charts showed the fold differences of cytokine expression in macrophages (A) without or (B) with shRNA-mediated knockdown of DJ-1, which were treated (IgG group) or not treated (control group) with 1 μg/ml isotype-control antibody for 24 hours. Data were presented as mean ± S.E.M. (n = 3 for each group). All data were normalized to levels of respective cytokines in control groups in order to calculate fold changes. Note that there is no difference.

In addition to IL-1β, other immunosuppressive cytokines were also detected in serum in DJ-1 KO mice. It is interesting to know whether macrophages serve as a potential source of several immunosuppressive cytokines, such as IL-4, IL-10, and TGF-β. We compared expressional profiles of the cytokines in macrophages with and without knockdown of DJ-1, and found that DJ-1 deficiency up-regulated IL-10, but has no effect on IL-4 and even down-regulated TGF-β in macrophage cells (Fig. 9), suggesting that macrophage-derived IL-10 might also contribute to build the local immunosuppressive microenvironment in lung tissue of DJ-1 knockout mice. This suggestion needs further investigation. In addition, macrophages can be activated by immunoglobulin complexes through Fc receptors to release IL-10 and IL-1β [38]. Since IL-1β neutralizing antibody was used in this study, we needed to check whether the free antibody can cause non-specific expression of the cytokines in macrophage through Fc receptors or not. Our additional experiments demonstrated that there was no difference in the expression of IL-1β, IL-4, IL-10, and TGF-β in macrophages without or with the presence of isotype-control antibody (Fig. 10), indicating that the anti-IL-1β antibody affect cancer development only through specific blockage of IL-1β, rather than through non-specific activation of macrophage.

A recent epidemiological research indicates that PD patients have higher risk of getting some specific cancers such as melanoma, but the reason is still unclear. It can be inferred that genes involved in the pathogenesis of PD might also participate in the development of cancers and thus contribute to the high risk of certain cancers in PD patients [8, 9]. For example, loss of function of PARK2, which encodes an E3 ubiquitin ligase called Parkin, has been known to be associated with development of both PD and cancer. PARK2 is therefore regarded as a tumor suppressor gene [39]. In contrast, DJ-1 is known as an oncoprotein and loss of DJ-1 is predicted to inhibit cancer growth, thus the loss of DJ-1 in PD patients is theoretically not a reason to contribute to cancer development. However, the present study clearly demonstrated that immunosuppressive microenvironment established in DJ-1 KO mice can increase migration of melanoma cells to lungs. Consistently, increased migration of lewis carcinoma to lungs was also noticed in DJ-1 KO mice (S1 Fig.). Thus, the risk of oncogenesis should be reconsidered in PD patients with DJ-1 deficiency.

**Fig 11:**
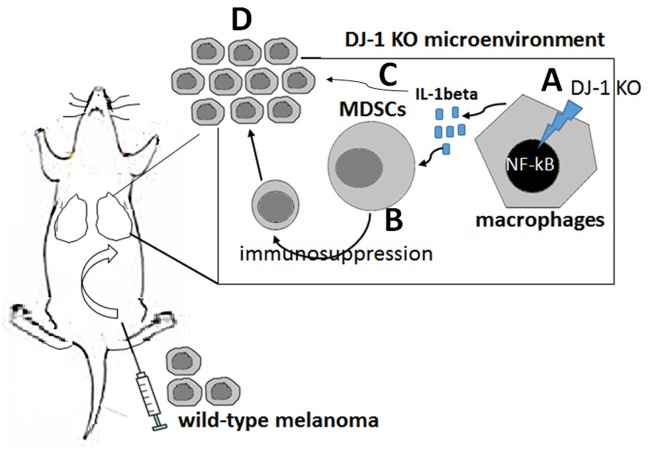
Schematic diagram of enhanced migration of cancer cells to lungs in DJ-1 KO mice. DJ-1 deficiency can (A) up-regulate IL-1β in macrophages through NF-κB pathway. The elevated IL-1β in DJ-1 KO mice then (B) recruits MDSCs to lungs to build an immunosuppressive microenvironment and/or (C) to enhance the metastatic ability of cancer cells, and, finally (D) promote cancer formation in lung tissue.

In summary (Fig. 11), our data demonstrated that DJ-1 deficiency can activate macrophages via NF-κB signaling to produce excess IL-1β, which is turn regulate MDSCs to build an immunosuppressive microenvironment for melanoma development. These findings may shed light on the mechanism of cancer development in DJ-1 associated PD patients.

## Supporting Information

S1 Fig Increase of lung-carcinoma nodules in DJ-1 KO mice. (A) LL/2 lewis lung carcinoma cells (6×10^4^) were intravenously injected into mice. The mice were sacrificed two weeks later. Gross images showed the lung-carcinoma nodules (arrows in upper panel), and histological images showed tumor masses (arrows in lower panel) in the WT and DJ-1 KO mice. Scale bar: 1 mm for photographs and 0.2 mm for H&E staining. (B) Bar chart showed the summarized results of lung nodules in WT and DJ-1 KO mice. Data are presented as mean ± S.E.M. * P<0.05 compared with WT mice. (n = 10 for each group, * P<0.05 compared with WT mice).
(TIF)
