# Supplementary figures and images for: Local Immunosuppressive Microenvironment Enhances Migration of Melanoma Cells to Lungs in DJ-1 Knockout Mice
Source: PLoS One. 2015 Feb 23;10(2):e0115827. doi: 10.1371/journal.pone.0115827 (PMC4338246; doi:10.1371/journal.pone.0115827)

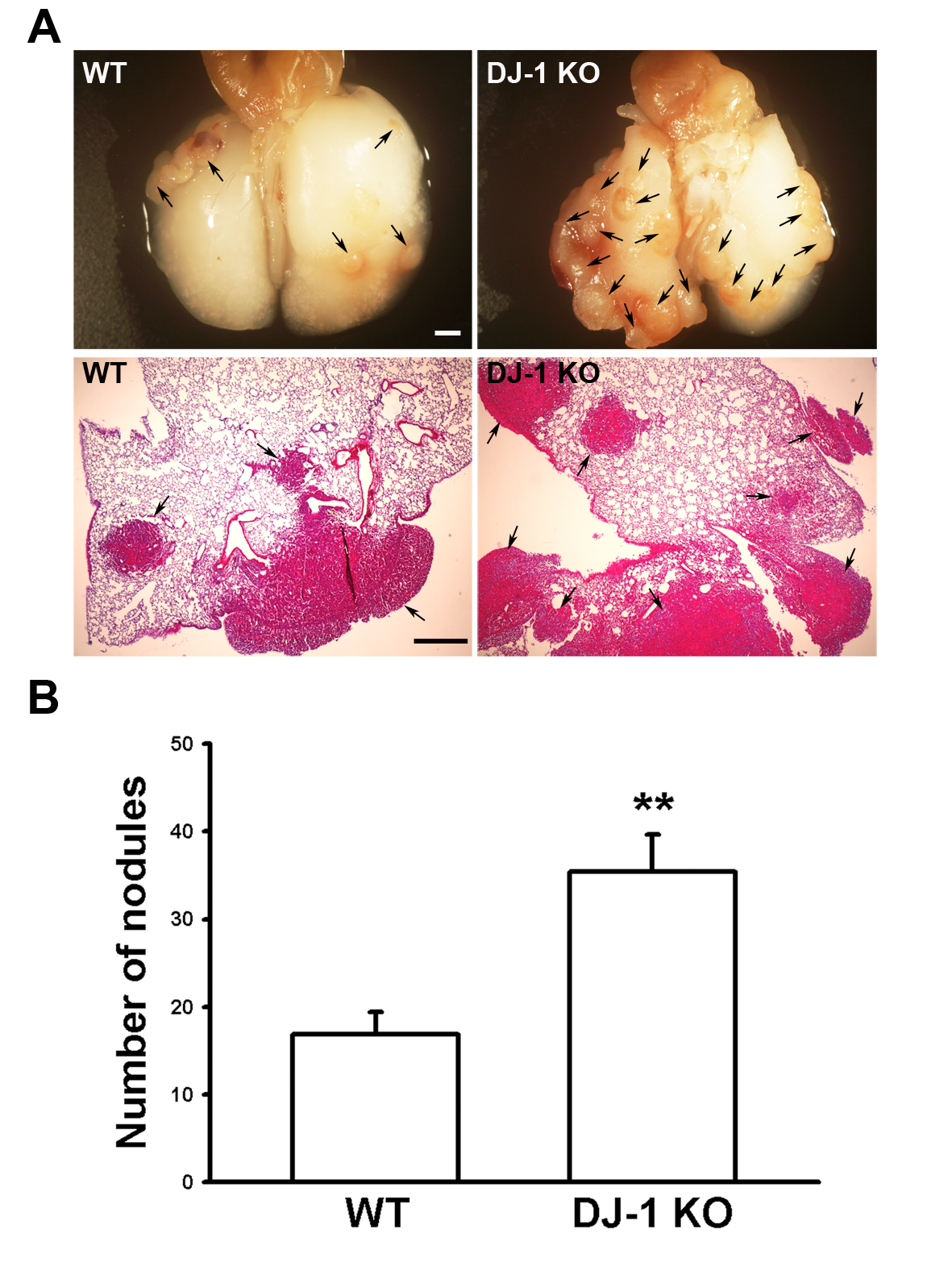

Supplement: S1 Fig — (A) LL/2 lewis lung carcinoma cells (6×10^4^) were intravenously injected into mice. The mice were sacrificed two weeks later. Gross images showed the lung-carcinoma nodules (arrows in upper panel), and histological images showed tumor masses (arrows in lower panel) in the WT and DJ-1 KO mice. Scale bar: 1 mm for photographs and 0.2 mm for H&E staining. (B) Bar chart showed the summarized results of lung nodules in WT and DJ-1 KO mice. Data are presented as mean ± S.E.M. * P<0.05 compared with WT mice. (n = 10 for each group, * P<0.05 compared with WT mice). (TIF) [file pone.0115827.s001.tif]
